# Simultaneous primary cancer occurrence of melanoma and pulmonary adenocarcinoma in leptomeningeal metastases: a case report

**DOI:** 10.1186/s12885-019-6183-2

**Published:** 2019-10-23

**Authors:** Ann-Kathrin Stoppek, Sied Kebir, Andreas Junker, Kathy Keyvani, Stefan Zülow, Lazaros Lazaridis, Teresa Schmidt, Daniela Pierscianek, Martin Stuschke, Ulrich Sure, Christoph Kleinschnitz, Björn Scheffler, Lisa Zimmer, Martin Glas

**Affiliations:** 1 Division of Clinical Neurooncology, Department of Neurology, University Hospital Essen, University Duisburg-Essen, Hufelandstr. 55, 45147 Essen, Germany; 2 West German Cancer Center (WTZ), University Hospital Essen, University Duisburg-Essen, Essen, Germany; 3 German Cancer Consortium, Partner Site University Hospital Essen, Hufelandstr. 55, 45147 Essen, Germany; 4 DKFZ Division of Translational Neurooncology at the West German Cancer Center (WTZ); German Cancer Consortium (DKTK), Partner Site University Hospital Essen, Hufelandstr. 55, 45147 Essen, Germany; 5 Institute of Neuropathology, University Hospital Essen, University Duisburg-Essen, Hufelandstr. 55, 45147 Essen, Germany; 6 Institute of Diagnostic and Interventional Radiology and Neuroradiology, University Hospital Essen, Hufelandstr. 55, 45147 Essen, Germany; 7 Department of Neurosurgery, University Hospital Essen, University Duisburg-Essen, Hufelandstr. 55, 45147 Essen, Germany; 8 Department of Dermatology, University Hospital, University Duisburg-Essen, Germany & German Cancer Consortium (DKTK), Heidelberg, Germany; 9 Department of Radiotherapy, University Hospital Essen, Essen, University Duisburg-Essen, Hufelandstr. 55, 45147 Essen, Germany; 10 Department of Neurology, University Hospital Essen, University Duisburg-Essen, Hufelandstr. 55, 45147 Essen, Germany

**Keywords:** Leptomeningeal metastasis, Adenocarcinoma, Melanoma, Simultaneous

## Abstract

**Background:**

Leptomeningeal metastasis (LM) is a predominantly late stage, devastating complication of a variety of malignant solid tumors. Diagnosis relies predominantly on neurological, radiographic, and cerebrospinal fluid (CSF) assessments. Recently, liquid biopsy tests derived from CSF has shown to be a feasible, noninvasive promising approach to tumor molecular profiling for proper brain cancer diagnostic treatment, thereby providing an opportunity for CSF-based personalized medicine. However, LM is typically misleadingly assumed to originate from only one primary tumor type.

**Case presentation:**

In this case report, we provide first evidence of the co-occurrence of LM originating from more than one primary tumor types.

**Discussion and conclusions:**

Based on this patient case profile, the co-occurrence of LM from two or more primary tumor types should be accounted for when deriving diagnostic conclusions from liquid biopsy tests.

## Background

LM results from spread or metastases of cancer cells to the leptomeninges, CSF, and the subarachnoid space [1, 2]. LM is detected in 9–25% of patients with lung cancer [3] and 6–18% of patients with melanoma [4]. The highest clinical diagnostic sensitivity is achieved by contrast-enhanced magnetic resonance imaging (MRI; cranial and complete spinal axis) in combination with CSF cytology evaluations. Treatment options are limited and include systemic and intrathecal medical therapy and radiotherapy (RTX) of the affected regions [2]. Intra-CSF and systemic therapies should be geared towards treatment algorithms that target primary tumor histology. LM prognosis is invariably poor and diagnosis usually occurs at an advanced disease stage and is associated with a high systemic tumor burden [2, 5]. Without treatment, LM of solid tumors, such as melanoma or lung cancer, typically lead to death within 4–6 weeks [6]. The objective of this case report is to describe first evidence of LM originating from the co-occurrence of 2 distinct primary tumor types known to be frequently associated with leptomeningeal disease spread.

## Case presentation

**Fig. 1:**
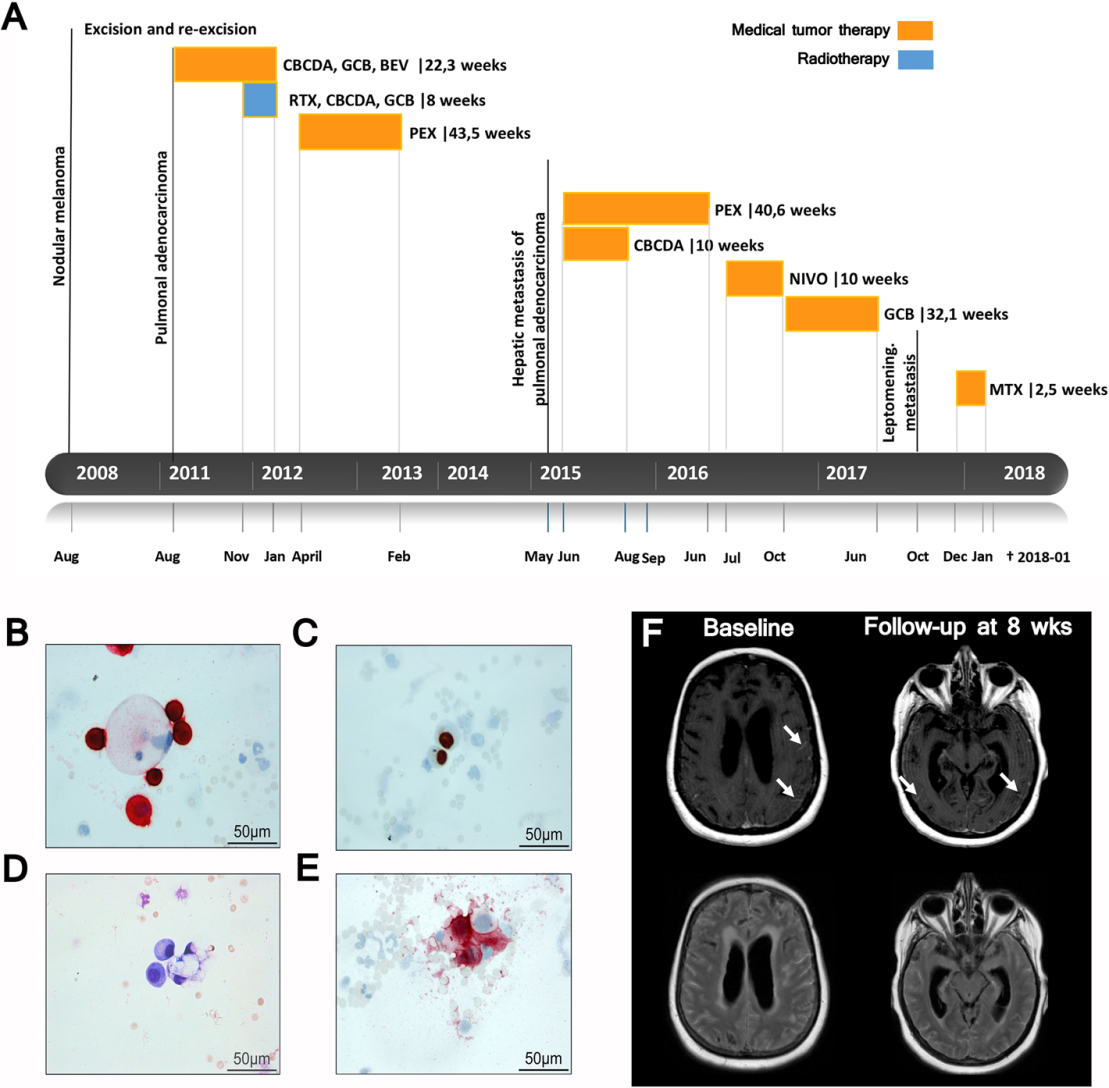
**a** Tumor-directed treatment over time schematic from melanoma diagnosis until time of death. **b**-**e** Immunohistological staining: **b** Melan A: 5 melanoma cells with intense red staining are shown that surround 1 carcinoma cell with no coloration. **c** TTF-1 expression is shown in pulmonary adenocarcinoma cells, but not in the melanoma cells. **d** May-Grünwald-Giemsa (MGG) staining: 2 melanoma cells and 1 adenocarcinoma cell with cytoplasmic inclusions. **e** HMB45 staining: red stained melanoma cells with 1 uncolored carcinoma cell is shown. **f** MRI scans of LM are shown in contrast-enhanced T1 sequence and T2-FLAIR at baseline and at 8-week follow-up visit. In comparison to baseline, follow-up scans showed LM disease progression. White arrows indicate LM in T1 scans. In the T2-FLAIR scans, hyperintensities tracking along the sulci clearly indicate LM

We describe the case of a 65-year-old Caucasian female with recurrent focal aware seizures (FAS; also known as simple partial seizures) who exhibited motor dysfunction of the right arm and leg as well as symptoms of nausea, regurgitation, and deafness in October of 2017. In 2008, the patient was diagnosed with nodular melanoma on her left arm (tumor thickness 0.6 mm, Clark Level IV, pT1aN0M0 stage IA; excised completely) and pulmonary adenocarcinoma (initial stage: cT2, cN3, cN0, M0, G3) in 2011 (Fig. 1a). From 2011 to 2012, the patient received chemotherapy (CTX: carboplatin, gemcitabine) and the antiangiogenic bevacizumab in addition to RTX (64.8 Gy local tumor region, 50.4 Gy mediastinum), followed by maintenance pemetrexed until 2013. In 2015, liver metastases were detected, during regular cancer staging examinations, and were histologically defined as metastases originating from pulmonary adenocarcinoma. CTX rechallenge with carboplatin and pemetrexed was initiated for 4 additional cycles. Computed tomography (CT) staging examinations of the thorax and abdomen confirmed stable disease. Relapse of liver metastases was detected in 2016 and treatment with nivolumab (NIVO; 270 mg Q2W) was initiated, however 4 months later hepatic and pulmonary metastases recurred. Systemic treatment was switched to gemcitabine monotherapy, and follow-up diagnostic imaging confirmed stable disease 6 months later. During a diagnostic routine work-up for recurrent FAS in October of 2017, an MRI of the brain and spine was conducted and raised concerns of the potential presence of LM. CT imaging of the thorax and abdomen determined that there was no extracerebral manifestation of tumor recurrence (Fig. 1a). However, the patient became increasingly disoriented and presented with emergent somnolence, as the primary clinical manifestation. CSF evaluation revealed moderate pleocytosis with leukocytes of 29/μl, total protein of 62 mg/dl, and lactate of 4.6 mg/dl; and showed pathohistological evidence of tumor cells of diverse morphology (Fig. 1b-e). More specifically, CSF cytology revealed 2 distinct tumor cell populations. Approximately 50% of the first tumor cell population showed enlarged hyperchromatic nuclei with broad, irregularly shaped basophilic cytoplasm. Secondly, the remaining half of tumor cells were characterized by enlarged hyperchromatic nuclei with a narrow band of irregular basophilic cytoplasm, cytoplasmic inclusions, and several signet rings. Inclusively, morphological assessment showed presence of both activated lymphocytes and monocytes. Immunocytochemical analysis showed that approximately half of the tumor cells stained positive for cytokeratin 8 and 7 (CK8, CK7) with nuclear staining to Thyroid Transcription Factor 1 (TTF1) (Fig. 1c) and cytoplasmic inclusions in May-Grünwald-Giemsa (MGG) staining (Fig. 1d) that were consistent with metastasis from pulmonary adenocarcinoma. The other half of cells showed slight CK8 positivity with strong positivity for melanosome antigens MelanA (Fig. 1b) and Human Melanoma Black 45 (HMB45) (Fig. 1e), which was consistent with metastasis from malignant melanoma. DNA analysis revealed the presence of wildtype BRAF, mutant tumor protein 53 (TP53), and KRAS tumor cells as detected by next-generation sequencing.

After placement of an Ommaya reservoir, we initiated (Dec 2017) intrathecal CTX with methotrexate (MTX; 12 mg administered in 3-day intervals) after which the patient was considerably more awake and orientated. On subsequent CSF analysis leukocytes and protein normalized from the first dose of MTX onward. There was no adverse event from MTX. After the sixth administration, the patient’s symptoms worsened in terms of increased disorientation, fatigue, and generalized motor weakness, while CSF analysis revealed pleocytosis (18/μl) and total CSF protein of 98 mg/dl. On follow-up, an MRI of the brain showed significant increases of signs consistent with supra- and infratentorial LM (Fig. 1f, g). As the patient’s clinical status worsened significantly, a tumor-directed treatment was terminated and the patient received best supportive care. The patient died 4 months after the cytological diagnosis of LM, 9.4 years from the diagnosis of melanoma and 6.4 years from the initial diagnosis of pulmonary adenocarcinoma. Written informed consent was obtained from the participant for the publication of this case report.

## Discussion and conclusions

To our knowledge, this is the first case report to present the simultaneous occurrence of LM from both a pulmonary adenocarcinoma and malignant melanoma.

In light of ever-increasing novel cancer therapeutics, particularly with the advent of immunotherapy, the likelihood of developing LM has grown substantially [7]. As such, clinicians might be more frequently confronted with similar cases, mainly when LM represents the primary tumor manifestation [8, 9]. Also, only recently has liquid biopsy of CSF been shown to be feasible, thereby facilitating precision medicine [10]. We herewith oppose the notion that LM cannot emerge from more than 1 tumor type at once. The possibility of co-occurrence of more than one primary tumor type in LM should be accounted for in any effort towards CSF-associated precision medicine. Regarding this case report, one might scrutinize whether the detected cells in the CSF truly represent melanoma cells given the unusual late presentation of metastasis and a lack of systemic melanoma progression. However, the cytomorphological appearance clearly supports the diagnosis of tumor cells as opposed to immune cells or non-malignant cells. The cytochemical presence of MelanA and HMB45 further underlines the melanocytic lineage. In conclusion, the presented findings can best be reconciled with LM melanoma.

Currently, there is no evidence-based pathophysiological explanation for the co-occurrence of LM from 2 different cancers. Still, it is tempting to speculate that impairment of the blood-brain barrier induced by 1 cancer might lead to a higher probability of another co-existing malignant disease to induce LM as well. As treatment decisions mainly rely on primary tumor histology, it is essential to determine from which tumor entity LM originated, especially in patients with prior history of 2 or more solid tumors known to evoke LM. This will become increasingly of relevance since novel tumor-specific systemic and intrathecal therapeutics are being designed and studied in phase 1 LM trials (ClinicalTrials.gov Identifier: NCT03025256) [11]. Therefore, it is essential for the treating physician to consider the rare possibility of LM originating from different tumor types in the same patient. To further our understanding of how LM develops, there is a need for prospective clinical and preclinical LM studies.

To our knowledge, this is the first case report to demonstrate the co-occurrence of LM from both a pulmonary adenocarcinoma and malignant melanoma. With the remarkable success concerning liquid biopsy from CSF in brain cancer, correctly detecting LM becomes more critical than ever.

## Data Availability

Dr. Sied Kebir (sied.kebir@uk-essen.de) should be contacted to request the data.

## Abbreviations

CSF: Cerebral spinal fluid
CT: Computed tomography
CTX: Chemotherapy
LM: Leptomeningeal metastasis
MGG: May-Grünwald-Giemsa
MRI: Magnetic resonance imaging
MTX: Methotrexate
